# Intraperitoneal ketamine versus bupivacaine for postoperative pain control after laparoscopic cholecystectomy – a double-blind randomized controlled trial

**DOI:** 10.1186/s12871-025-03386-3

**Published:** 2025-10-14

**Authors:** Shehrum Bughio, Syed Muhammad Nadeem, Arsala Rahman, Palwasha Khan Kasi, Sadam Hussain, Hanesh Tanwani

**Affiliations:** Department of Anesthesiology, Critical Care, and Pain Management, Liaquat National Hospital & Medical College, National Stadium Road, Karachi, Pakistan

**Keywords:** Laparoscopic surgery, Ketamine, Bupivacaine, Intraperitoneal instillation, Cholecystectomy

## Abstract

**Background:**

Intraperitoneal instillation of local anesthetics is commonly used to manage pain associated with laparoscopic surgeries. Few studies have compared intraperitoneal instillation of ketamine with bupivacaine for postoperative pain control in patients undergoing laparoscopic cholecystectomy (LC). This study aimed to compare the effects of intraperitoneal instillation of ketamine versus bupivacaine on postoperative pain control following LC.

**Methods:**

This double-blind randomized controlled trial was conducted by the Department of Anesthesiology, Critical Care, and Pain Management at Liaquat National Hospital. Patients were divided into Group K and Group B. Group K received ketamine at a dose of 0.25 mg/kg diluted in 40 ml of saline intraperitoneally, while Group B received 2 mg/kg bupivacaine diluted in 40 ml of saline. Pain scores were assessed using the Numeric Rating Scale at 5 min, 15 min, 6 h, 12 h, and 24 h postoperatively.

**Results:**

A total of 46 patients in each group were studied. At 5 min, the average pain score was 2.1 ± 0.5 in Group K and 2.8 ± 0.7 in Group B (*p* < 0.001). At 15 min (1.5 ± 0.2 versus 3.6 ± 0.4), 6 h (2.5 ± 0.5 versus 4.8 ± 0.4), 12 h (3 ± 1.1 versus 4.5 ± 0.7), and 24 h (1.8 ± 0.8 versus 4.1 ± 1.2), the mean pain score was significantly lower in Group K than in Group B. Analgesia requirement was significantly higher in the bupivacaine group, whereas the mean time to seek the first rescue analgesia was longer in the ketamine group. No adverse events other than postoperative nausea and vomiting were observed.

**Conclusion:**

The present study found that in elective LC, intraperitoneal instillation of ketamine at a dose of 0.25 mg/kg was superior to bupivacaine alone in reducing postoperative pain and analgesic demand from initial 6 to 24 h after surgery, without any safety concerns.

**Clinical trial number:**

This trial was retrospectively registered on January 29, 2025, in clinicaltrials.gov with trial number ‘NCT06807554’.

## Background

Laparoscopic cholecystectomy (LC) is the standard treatment for cholelithiasis, but it is also associated with postoperative pain [1]. Despite small port site incisions, postoperative pain management remains a challenge that impacts patient comfort, recovery, the possibility of same-day discharge, and overall satisfaction [2].

Visceral pain, somatic pain, and transferred discomfort (shoulder soreness) are all brought on by LC. Shoulder pain is caused by carbonic acid, which is created when carbon dioxide and water combine, irritating the phrenic nerve; visceral discomfort is caused by the straining of internal organs during carbon dioxide inhalation. The cut pain caused by the ports is linked to somatic discomfort. Post-cholecystectomy syndrome (PCS), which has been found to affect 12% to 40% of patients, may be brought on by severe postoperative discomfort [3–6].

Various strategies are employed to manage post-LC pain, including wound infiltration, regional anesthesia, neuroaxial analgesia, patient-controlled analgesia, and systemic opioid and non-opioid analgesics. Although ultrasound-guided regional blocks are being used widely for supplemental analgesia in LC, the cost of ultrasound machines limits their use in resource-limited areas [7]. Use of local anesthetic (LA) infiltration as part of multimodal analgesia can help reduce postoperative pain and analgesic consumption, potentially promoting faster recovery and shorter hospital stays [8, 9].

Intraperitoneal local anesthetic (IPLA) administration has been shown to effectively manage post-LC pain, primarily caused by pneumoperitoneum [10]. Ketamine, an NMDA receptor antagonist, has potent analgesic properties at sub-anesthetic doses (0.1–0.5 mg/kg IV) [11]. Studies have demonstrated the efficacy of ketamine instillation on the gallbladder bed in reducing postoperative pain [12, 13].

Bupivacaine, an amide local anesthetic, is commonly used for pain control at port site incisions [14]. Its mechanism of action involves blocking sodium channels, thereby inhibiting pain transmission [15]. Bupivacaine is preferred for its selective effect on sensory nerve fibers [16].

Effective pain management enhances postoperative outcomes, including early mobilization and reduced pulmonary complications, deep vein thrombosis, and pulmonary embolism. This study aimed to compare the efficacy of intraperitoneal ketamine versus bupivacaine for postoperative pain control after LC.

## Materials and methods

### Study design and settings

This double-blind randomized controlled trial was conducted in the Department of Anesthesiology, Critical Care, and Pain Management at Liaquat National Hospital and Medical College, Karachi, Pakistan.

### Inclusion & exclusion criteria

The study included ASA I and II patients, aged 20 to 70 years, of either gender, who were scheduled for elective laparoscopic cholecystectomy (LC). Exclusion criteria were known allergies to the study drugs, chronic pain conditions, those on chronic pain medications, psychiatric illnesses, those weighing more than 80 kg (for avoiding sub-anesthetic dose or exceeding the maximum safe dose of 150 mg of bupivacaine), patients requiring emergency surgery, or those converted to open cholecystectomy.

### Sample size estimation

A sample size of 46 per group (1:1 ratio) was calculated using the Open-Epi online calculator, based on the mean VAS (visual analogue scale) score of 3.0 ± 0.86 for the ketamine group and 3.50 ± 0.84 [17] for the bupivacaine group at 12 h. The effect size was 0.588, power was 80%, and the confidence interval was kept at 95% in the sample size calculation. Patients were enrolled using consecutive sampling, while groups were randomly assigned through opaque, sealed, consecutively numbered envelopes.

### Preoperative assessment and group assignment

Patients scheduled for LC were informed about the study during their pre-anesthesia assessment, and their written informed consent was obtained. After pre-anesthesia assessment, patients were either admitted to the ward the day before surgery or on the same day, where patients were reviewed again. A six-hour nil per os (NPO) restriction was advised before surgery. In the preoperative holding area, patients were allocated to either of two study groups by randomization employing a sequentially numbered opaque sealed envelope (SNOSE) protocol. The envelopes were labeled by the principal investigator and kept safely with the study’s noninvolved nursing staff. Each envelope was opened only after the participant’s eligibility was verified and consent was secured, thus ensuring allocation concealment. The residents involved, who were blinded to the order, recruited participants and carried out the intervention accordingly. Group K was given intraperitoneal ketamine, and Group B was given intraperitoneal bupivacaine.

### Anesthesia protocol

General anesthesia was given based on hospital protocol. No premedication was given. After establishing intravenous access and ASA standard monitoring, general anesthesia was induced with nalbuphine 0.15 mg/kg, propofol 2 mg/kg, and cisatracurium 0.15 mg/kg. Asthmatic patients received rocuronium (0.6 mg/kg) instead of cisatracurium. Anesthesia was maintained with isoflurane, oxygen, and air. Acetaminophen 15 mg/kg (up to 1 g) was given after induction, and all patients in both groups received 1 g of acetaminophen IV every 8 h postoperatively.

### Drug preparation, blinding and administration

The study drugs were prepared by an independent anesthetist who was not involved in the study and was unaware of the group assignments. Similarly, the surgeon administering the injections was also blinded to the specific drugs, and patients also had no knowledge of the drugs they received. Residents assessing study outcomes were kept blinded to the actual group allocations.

The surgery was performed by a consultant surgeon with at least 5 years of relevant experience. In Group K, ketamine (0.25 mg/kg) was diluted in normal saline to a total volume of 40 ml. The surgeon instilled 20 ml before starting the gallbladder dissection over the surgical site and the remaining 20 ml at the end of the procedure after securing hemostasis over the gallbladder bed. In Group B, bupivacaine (2 mg/kg) was diluted in normal saline to a total volume of 40 ml and administered in the same manner as in Group K. The bupivacaine dosage of 2 mg/kg was within the recommended safety margins for simple bupivacaine because it was less than the maximum total dose of 150 mg per patient. Injection of IV ondansetron 4 mg was administered before the end of surgery for prevention of postoperative nausea and vomiting. The anesthesia resident who prepared the medications for both groups was not involved in the postoperative pain evaluation, and another anesthesia resident assessed the pain who was unaware of the study drugs administration.

### Postoperative assessment and management and data collection

Pain assessments and monitoring was for adverse events were done postoperatively at 5 min, 15 min, 6 h, 12 h, and 24 h postoperatively. Tramadol (1.5 mg/kg, up to 100 mg IV) was given as rescue analgesia. If the NRS (Numeric Rating Scale) was 4 or higher and tramadol was inadequate, nalbuphine (0.1 mg/kg IV) was added.

### Study outcomes

The primary outcome of the study was “pain.” The secondary study outcome was “any adverse event because of study drugs.”

### Data collection and definition of study variables

Patient demographic information, including age, gender, body mass index (BMI), comorbidities, ASA grade, surgery duration, and pain status, was collected. Surgery duration was defined as the time from incision to wound closure. Pain was assessed using a numeric rating scale (NRS), with 0 indicating no pain and 10 representing the worst possible pain. Shoulder pain was regarded as any discomfort in the shoulder area. Visceral pain was described as spontaneous, dull, poorly localized, deep-seated abdominal pain. Somatic pain was defined as sharp pain at port sites in the abdominal wall. If NRS was greater than 3, the time to first rescue analgesia was noted. The total amount of analgesics used in the first 24 h after LC was documented. As a secondary outcome, adverse events because of medications administered were also monitored and recorded.

### Statistical analysis

Statistical analysis was performed using IBM SPSS version 27. Frequencies and percentages were used for categorical variables. The Shapiro-Wilk test was used to check for normality of numerical variables. For normally distributed numerical data, mean ± standard deviation was reported. The chi-square or Fisher-exact test was used to compare categorical variables between the two groups. The independent t-test was applied to compare numerical variables. A p-value of < 0.05 was considered statistically significant.

## Results

**Fig. 1:**
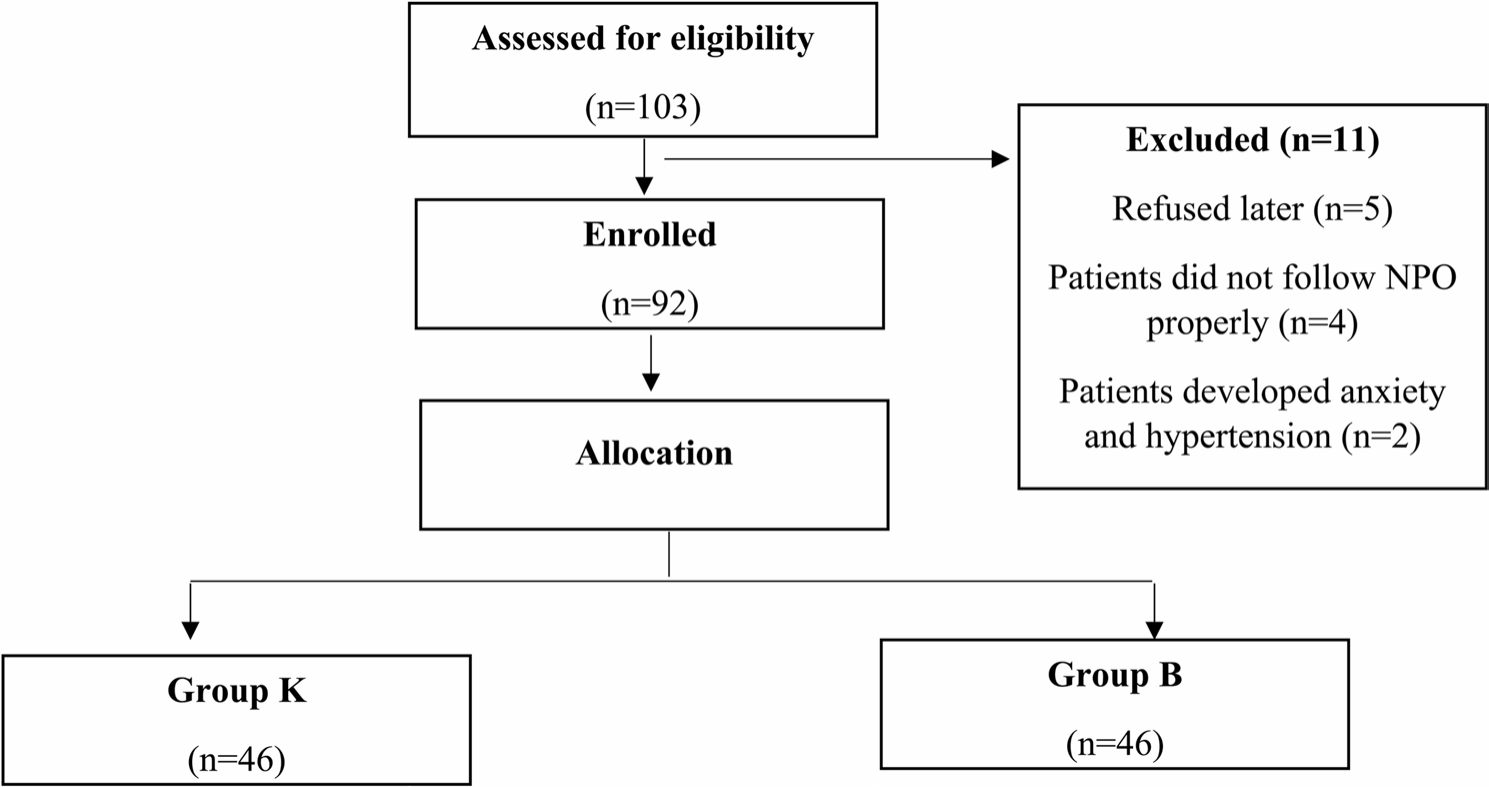
Consort diagram of study

Initially 103 patients were enrolled, but 11 were excluded. Finally, a total of 92 patients were studied, with 46 patients in each arm (Fig. 1). Two groups did not differ on the basis of age, gender, height, weight, BMI, ASA grade, and surgery duration (Table 1). No intraoperative complications were observed.

**Table 1:** Comparison of patients’ sociodemographic and clinical profile among study groups

Variables	Group K	Group B	*p*-value
Age (years), mean ± SD	45.7 ± 13.2	46.4 ± 12.9	^ϕ^0.806
Gender, n(%)			
Male	23(50)	22(47.80)	^€^0.835
Female	23(50)	24(52.2)	
Weight (Kg), mean ± SD	74.7 ± 16.57	74.61 ± 16.94	^ϕ^0.832
Height (inches), mean ± SD	161.83 ± 8.41	161.89 ± 9.21	^ϕ^0.971
Body mass index (Kg/m^2^), mean ± SD	28.7 ± 5.8	28.4 ± 6.1	^ϕ^0.749
ASA grade, n(%)			
ASA I	28(60.9)	26(56.5)	^€^0.672
ASA II	18(39.13)	20(43.5)	
Surgery duration (minutes), mean ± SD	71.5 ± 2.48	71.85 ± 2.47	^ϕ^0.614

*ASA* American Society of Anesthesiologists, *SD* Standard deviation

^ϕ^Independent t-test was applied for numerical data

^€^Pearson Chi-square test has been applied for categorical data

**Fig. 2:**
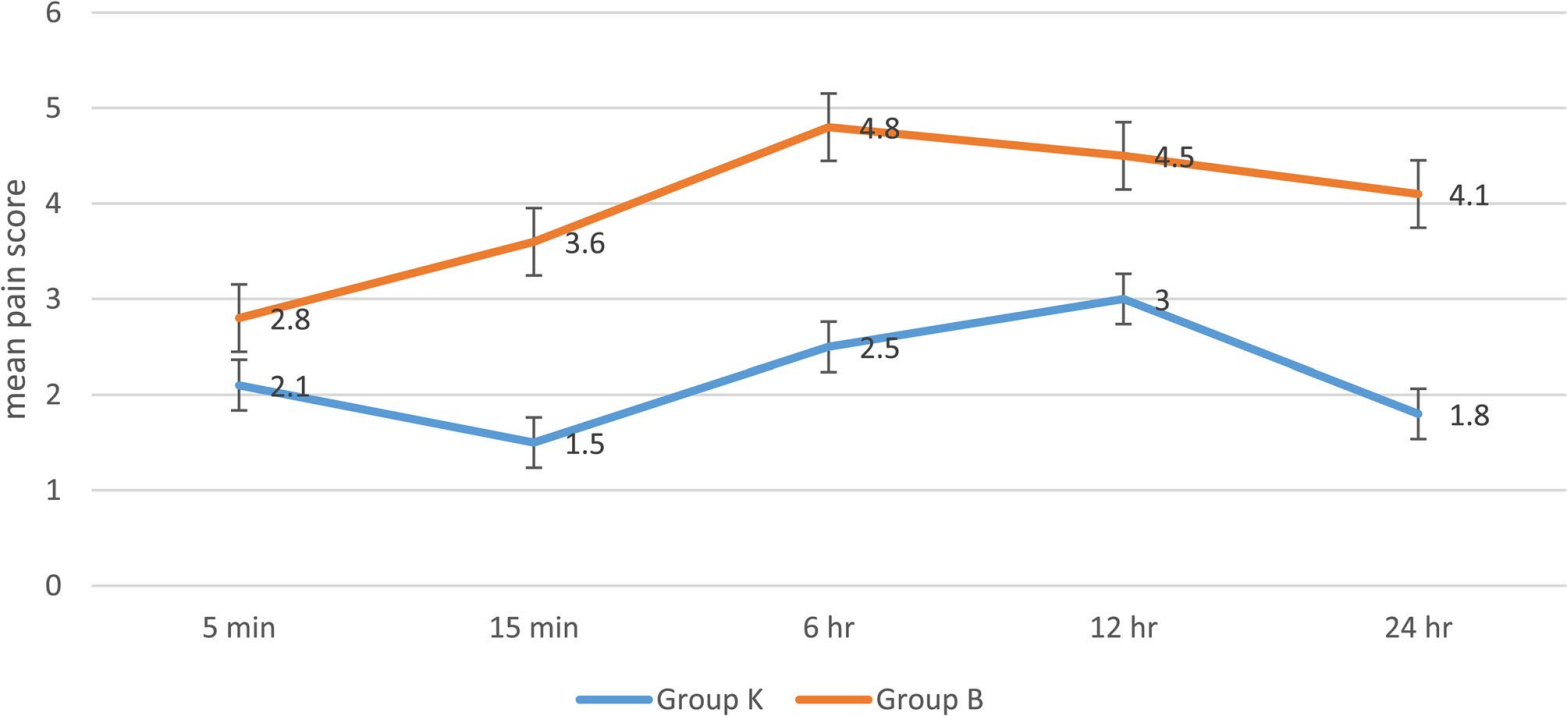
Comparison of pain score among the two study groups

Figure 2 displays a comparison of the mean score from 5 min to 24 h of the procedure. At 5 min, the average pain score was 2.1 ± 0.5 in Group K and 2.8 ± 0.7 in Group B (*p* < 0.001) mean difference and effect size of −0.7 ± 0.3 and 1.15 respectively. At 15 min, the mean score was 1.5 ± 0.2 and 3.6 ± 0.4 in Group K and Group B (*p* = 0.002) respectively. At 15 min, mean difference and effect size − 2.1 ± 0.1 and 6.6 respectively. At 6 h (2.5 ± 0.5 compared to 4.8 ± 0.4, mean difference: −2.3 ± 0.2, Cohen’s d: 5.1, *p* < 0.001), 12 h (3 ± 1.1 versus 4.5 ± 0.7, mean difference: −1.5 ± 0.4, Cohen’s d: 1.5, *p* < 0.001), and 24 h (1.8 ± 0.8 versus 4.1 ± 1.2, mean difference: −2.3 ± 0.4, Cohen’s d: 2.2, *p* < 0.001), the mean pain score was considerably lower in Group K than in Group B.

Table 2 compares the shoulder, visceral, and somatic pain. Shoulder pain was significantly lower in Group K at 12 h and 24 h than in Group B. Visceral pain was found to be significantly different among both the groups, with a lower mean pain score in Group K from 6 h to 24 h. Somatic pain was not significantly different between the two groups throughout the study.

**Table 2:** Comparison of NRS for different pain sites

Variables	Group K	Group B	^ϕ^*p*-value
Shoulder pain, mean ± SD			
5 min	0	0	-
15 min	1.1 ± 0.2	1.8 ± 0.8	0.741
6 h	1.8 ± 0.5	2.5 ± 1.2	0.296
12 h	1 ± 0.1	3.1 ± 1.6	*0.018
24 h	0 ± 0.7	1.2 ± 0.6	*0.006
Visceral pain, mean ± SD			
5 min	0 ± 0.2	1 ± 0.3	0.826
15 min	1 ± 0.4	2.1 ± 0.8	0.413
6 h	1.8 ± 0.7	2.8 ± 1.4	*0.029
12 h	1 ± 0.1	3 ± 0.8	*0.008
24 h	1.2 ± 0.6	3.2 ± 1.5	*0.001
Somatic pain, mean ± SD			
5 min	2.2 ± 0.5	2.5 ± 0.2	0.914
15 min	1 ± 0.8	2.2 ± 0.9	0.542
6 h	2.5 ± 1.3	3 ± 1.3	0.172
12 h	2.6 ± 1.1	3 ± 1.5	0.248
24 h	2 ± 1.5	3 ± 1.1	0.136

*SD* Standard deviation

*Significant at p < 0.05

^ϕ^Independent t-test was applied for numerical data

Table 3 compares how long it took for each group to request their first analgesia and how much analgesia they took overall in the first 24 h. The total amount of analgesia taken in the first 24 h was significantly higher in the bupivacaine group with mean difference and effect size of −20.9 ± 0.4 and − 13.4 respectively. The mean time to seek first analgesia was significantly higher in the ketamine group. The mean difference and effect size was 9.3 ± 0.5 and 6.4 respectively.

**Table 3:** Comparison of analgesic status among the two study groups

Variables	Group K	Group B	^ϕ^*p*-value
Time to first rescue analgesia (hours), mean ± SD	16.7 ± 1.2	7.5 ± 1.4	*<0.001
Total dose of rescue analgesia in 24 h (mg), mean ± SD	24.1 ± 1.2	45.0 ± 1.8	*<0.001

*SD* Standard deviation

*Significant at *p* < 0.05

^ϕ^Independent t-test was applied for numerical data

Table 4 displays a comparison of adverse events between the two study groups. Postoperative nausea and vomiting were seen in both groups, but they did not reach statistical significance. Other than postoperative nausea and vomiting, no adverse event was seen among both of the groups.

**Table 4:** Comparison of adverse events among both of the study groups

Variables	Group K	Group B	^€^*p*-value
Nausea, n(%)	6(13)	8(17.4)	0.245
Vomiting, n(%)	10(21.7)	13(28.3)	0.182

^€^Pearson Chi-square test has been applied for categorical data

## Discussion

Various perioperative analgesia techniques, such as systemic opioids, non-steroidal anti-inflammatory drugs (NSAIDs), LA wound infiltration, and neuraxial administration of LA and opioids, have been studied to reduce postoperative pain after LC. One such technique is intraperitoneal instillation of analgesic drugs, also used in other minimally invasive surgeries, like gynecological and laparoscopic bariatric surgery [18–21]. This method is effective because it blocks visceral nociceptive transmission and provides an additional mode of analgesia. Another possible mechanism could be absorption of the drug from the peritoneal surface [22].

Our study showed better postoperative pain control in the ketamine group compared to the bupivacaine group. Moharari RS et al. [17] found that pain control was better in the ketamine group during the early postoperative period (5 min to 6 h). Ketamine reduces central sensitization and pain by acting as an NMDA receptor antagonist, altering pain perception in the brain and spinal cord [12]. Bupivacaine, on the other hand, blocks sodium channels to stop nerve conduction at the injection site, with effects limited to specific areas [14]. Ketamine may be more useful for complex pain due to its ability to affect both peripheral and central pain pathways. Its superior pain control was also observed in other studies, including intrathecal administration [23]. Panigrahi et al. [23] found that the ketamine group experienced longer-lasting analgesia than the bupivacaine group after lower leg and lower abdomen surgeries.

Several studies have shown that intraperitoneal bupivacaine is more effective than a control group [5, 24, 25]. Nupur et al. [24] and Sharma et al. [25] found that bupivacaine reduced discomfort within the first 24 h after LC. Manan A et al. [5] found that bupivacaine controlled pain better than saline from 30 min to 12 h after surgery. Other studies have compared ketamine and bupivacaine mixtures to bupivacaine alone in LC patients [26, 27]. Mostafa RH et al. [26] found that the combination of ketamine and bupivacaine reduced postoperative shoulder pain and analgesic requirements more than bupivacaine alone. Ziaei P et al. [27] found that the combination provided better pain control than bupivacaine alone, but bupivacaine was more effective than the control group. This suggests ketamine enhances pain control due to its unique properties. It can be noted that with intraperitoneal administration, ketamine might have had systemic action through the NMDA receptor blockade. These central actions could have resulted in the observed analgesia. However, ketamine was found to have better pain control than bupivacaine, validating its utility in postoperative analgesia.

In our study, shoulder tip pain and visceral pain were lower in the ketamine group compared to the bupivacaine group. No difference was seen in somatic pain. This agrees with Mostafa RH’s study [26], which found lower shoulder and visceral pain in the ketamine-bupivacaine combination group. Mohrari et al. [17] reported only overall pain scores and did not focus on the three pain components. Somatic pain was similar in both groups, likely due to lower systemic absorption.

We also found that the time to first analgesic request was significantly longer in the ketamine group (16.7 ± 1.2 vs. 7.5 ± 1.4 h). This is consistent with other studies, though some reported longer times for ketamine. Mohrari et al. [17] and Mostafa RH [26] found mean times of 21.43 ± 0.50 h and 20.26 ± 0.835 h. The shorter time to first analgesia request in our study may be due to the lower ketamine dose (0.25 mg/kg), compared to 0.5 mg/kg used by others. Some studies with 0.5 mg/kg also reported shorter times, such as Shawky et al. [12] with 3.95 ± 1.05 h and Oza V et al. [12] with 6.73 ± 1.3 h. The shorter time to first analgesic request for the ketamine group as compared to other studies could be possibly due to differences in surgical technique, operative time, or different ketamine doses.

In line with existing literature, our study found lower analgesic consumption in the ketamine group than in the bupivacaine group. Both groups had similar rates of adverse events, with postoperative nausea and vomiting reported in both, but no significant differences, consistent with previous studies [17, 27].

The present study suffers from some limitations. The study was based on the evaluation of only pain control and did not evaluate pain status in the first 1–2 h. Besides pain control, hemodynamic status was not studied. Since we used the ketamine at a dose of 0.25 mg/kg, so we did not systematically assess potential psychiatric impacts such as hallucinations or dysphoria. Moreover, the study was performed in a single center with a limited sample size, due to which findings cannot be generalized. A future study may be performed addressing the limitations of the current study for validating our findings.

## Conclusion

The present study found that in elective LC, intraperitoneal instillation of ketamine at a dose of 0.25 mg/kg was superior to bupivacaine alone in reducing postoperative pain and analgesic demand from initial 6 to 24 h after surgery, without any safety concerns.

## Data Availability

The dataset used in this study will be available from corresponding author upon a reasonable request.

## Abbreviations

ASA: American Society of Anesthesiologists
BMI: Body mass index
IPLA: Intraperitoneal local anesthetic
LA: Local anesthetic
LC: Laparoscopic cholecystectomy
NMDA: N-methyl-D-aspartate
NPO: Nil per oral
NRS: Numeric Rating Scale
NSAIDs: Non steroidal anti-inflammatory drugs
PCS: Post-cholecystectomy syndrome
VAS: Visual analogue scale
